# Certain Killer Immunoglobulin-Like Receptor (KIR)/KIR HLA Class I Ligand Genotypes Influence Natural Killer Antitumor Activity in Myelogenous Leukemia but Not in Acute Lymphoblastic Leukemia: A Case Control Leukemia Association Study

**DOI:** 10.4274/tjh.galenos.2019.2019.0079

**Published:** 2019-11-18

**Authors:** Viktoria Plamenova Varbanova, Snejina Mihailova, Elissaveta Naumova, Anastasiya Petrova Mihaylova

**Affiliations:** 1 Military Medical Academy, Multiprofile Hospital for Active Treatment, Clinic of Hematology, Sofia, Bulgaria; 2 University Hospital Alexandrovska - Clinic of Clinical Immunology and Stem Cell Bank, Medical University, Sofia, Bulgaria

**Keywords:** Chronic myeloid leukemia, Acute lymphoblastic leukemia, Acute myeloblastic leukemia

## Abstract

**Objective:**

Natural killers (NK) cell function is mainly controlled by the expression of killer immunoglobulin-like receptors (KIRs) and their ligation with the corresponding ligands. The objective of this study was to investigate the putative association of KIRs, HLA class I ligands, and KIR/ligand combinations with rates of development of acute lymphoblastic leukemia (ALL), acute myeloid leukemia (AML), and chronic myeloid leukemia (CML).

**Materials and Methods:**

The KIR/HLA I genotypes of 82 patients with leukemia (ALL, n=52; AML, n=17; and CML, n=13) were determined by PCR-SSP method and compared with genotypes of healthy controls (n=126).

**Results:**

KIR genotype frequency differed significantly between myelogenous leukemia patients and healthy controls for KIR2DL5A (17.6% vs. 47.7%, p=0.02), KIR3DS1 (17.6% vs. 47.6%, p=0.02), and KIR2DS4*001 (36.6% vs. 20.2%, p=0.017). The incidence of homozygous HLA-BBw4 (31.0% vs. 12.5%, p=0.042) and HLA-Bw4Thr80 Thr80 (13.0% vs. 1.2%, p=0.01) was significantly elevated in myeloid leukemia patients compared to healthy controls. KIR/HLA class I ligand profile KIR3DS1(+)/L (-) was decreased and KIR3DL2(+)/HLA-A3/11(-) was increased among myeloid leukemia cases compared to controls.

**Conclusion:**

These data suggest that the activity of NK cells as determined by inherited KIR/HLA class I ligand polymorphisms influences the susceptibility to myelogenous leukemia, but not to lymphoblastic leukemia. Additionally, the KIR genotype characterized by the absence of the inhibitory KIR2DL2 and the activating KIR2DS2 and KIR2DS3 (ID2) was found at a lower frequency in patients compared to controls, which confirmed the need for complex analysis based on all possible KIR/HLA class I ligand polymorphism combinations.

## Abstract (tr)

**Amaç:**

Doğal öldürücü (NK) hücre fonksiyonu temel olarak öldürücü immünoglobulin-benzeri reseptör (KIR) yüzey ifadesi ve bunların ilgili liganda bağlanması ile ilişkilidir. Bu çalışmanın amacı KIR, HLA sınıf I ligandlar ve KIR/ligand ilişkisinin akut lenfoblastik lösemi (ALL), akut myeloid lösemi (AML) ve kronik myeloid lösemi (KML) oluşumu ile ilişkisini araştırmaktır.

**Gereç ve Yöntemler:**

Seksen iki lösemi hastasının (ALL, n=52; AML, n=17; ve KML, n=13) KIR/HLA I genotipleri PCR-SSP metodu ile çalışıldı ve sağlıklı kontrollerin (n=126) genotipleri ile karşılaştırıldı.

**Bulgular:**

KIR genotip frekansı myeloid lösemi hastaları ve sağlıklı kontroller arasında KIR2DL5A (%17,6 vs. %47,7, p=0,02), KIR3DS1 (%17,6 vs. %47,6, p=0,02), ve KIR2DS4*001 (%36,6 vs. %20,2, p=0,017) açısından belirgin farklılık gösterdi. Homozigot HLA-BBw4 (%31,0 vs. %12,5, p=0,042) ve HLA-Bw4Thr80 Thr80 (%13,0 vs. %1,2, p=0,01) sıklığı da myeloid lösemi hastalarında sağlıklı kontrollere göre belirgin olarak daha yüksekti. Kontrollerle karşılaştırıldığında myeloid lösemi hastalarında KIR/HLA sınıf I ligand profili olarak KIR3DS1(+)/L(-) azalmış ve KIR3DL2(+)/HLA-A3/11(-) artmış olarak bulundu.

**Sonuç:**

Bu bulgular, kalıtılan KIR/HLA sınıf I ligand polimorfizmleri ile belirlenen NK hücre aktivitesinin myeloid lösemiye yatkınlığı etkilediği ancak lenfoid lösemi yatkınlığını etkilemediğini düşündürmektedir. Ayrıca inhibitor KIR2DL2, aktivatör KIR2DS2 ve KIR2DS3 (ID2) ile karakterize KIR genotipi, hastalarda kontrollere oranla daha düşük bulundu, bu da bütün olası KIR/HLA sınıf I ligand polimorfizmlerine dayanan kompleks analizlerin gerekliliğini desteklemektedir.

## Introduction

In 2015, 4062 patients with leukemia were treated and/or monitored in the Republic of Bulgaria [1]. Similarly to other neoplastic diseases, the precise etiopathogenetic mechanism that leads to the transformation of “normal” hematopoietic cells into blast cells has not yet been elucidated. The idea of a combined influence of external environmental factors and “internal” factors in the onset and development of malignancies is increasingly being discussed.

NK cells play an important role in antitumor immune defense [2,3]. Their function is controlled by the interaction of cell surface receptors with appropriate ligands, among which the most important and best studied are killer immunoglobulin-like receptors (KIRs) and their HLA class I ligands [4,5,6,7,8]. Seventeen KIR genes and pseudogenes have been described so far [4]. KIRs are named based on the number of their extracellular Ig-like domains (2D or 3D) and by the length of their cytoplasmic tail (long [L], short [S], or pseudogene [P]) [9]. Ligands for most KIRs are specific patterns of the HLA class I molecules [6,7,8,9,10]. HLA C molecules with amino acid residues Ser and Asn at positions 70 and 80, respectively (Ser77 and Asn80), form the HLA-C1 KIR ligand group, which specifically binds KIR2DL1 and KIR2DS2. The HLA-C2 KIR ligand group (Asn77 and Lys80) interacts with KIR2DL2/2DL3 and KIR2DS1 [5,6,10,11,12,13]. HLA-B class I molecules with the Bw4 epitope are ligands for KIR3DL1 and KIR3DS1 [7,8,14,15]. The strength of the KIR/ligand binding is determined by the amino acid residue at position 80 in the Bw4 molecule (Bw4^Ile80^ are stronger ligands for their specific KIRs than Bw4^Thr80^) [7,8]. Data suggest that HLA-A alleles with Bw4 epitopes may be ligands for KIR3DL1 [11]. KIR3DL2 specifically recognizes HLA-A3 and HLA-A11 specific to certain peptides such as Epstein-Barr virus peptides [16,17].

The first immunogenic studies investigating the effect of KIRs and their HLA class I ligands and their relation to the development of oncohematological diseases were conducted by Demanet et al. [18] and Verheyden et al. [19] in 2004. There are increasing data on the association of genetic polymorphisms of KIRs and their HLA ligands with a predisposition to various hematological malignancies, although a specific polymorphism clearly associated with leukemia development has not been identified [20]. Indirect evidence for the role of NK cells in leukemia defense includes the proved decreased incidence of relapse and increased leukemia-free surveillance in the setting of allogenic KIR HLA class I ligand donor-recipient incompatibility hematopoietic stem cell transplantation [21,22]. These data additionally suggest that NK cells may play a major role in the control and clearance of leukemia. It seems that the interplay between the inhibitory KIR signals and/or the predominance of activating ones is critical for the NK-mediated anti-leukemic effect. Therefore, in the present study, we studied the polymorphism of NK-cell receptors, namely KIRs and their HLA class I ligands, in patients with leukemia and healthy controls and investigated the possible association of these immunogenic factors with different leukemias in the Bulgarian population.

## Materials and Methods

### Study Groups

Eighty-two patients with primary leukemia and 126 healthy controls were included after they provided signed informed consent. The healthy individuals were randomly selected from unrelated volunteers from the Bulgarian population, without chronic diseases and with negative family history of hereditary diseases, autoimmune diseases, or malignancies (46.1% male and 53.9% female, mean age 46.6±11.9 years). The patient group consisted of patients with acute lymphoblastic leukemia (ALL) (n=52, 65.4% male and 34.6% female, mean age 20.8±12.5 years), acute myeloid leukemia (AML) (n=17, 64.7% male and 35.3% female, mean age 36.9±11.3 years), and chronic myeloid leukemia (CML) (n=13, 84.6% male and 15.4% female, mean age 36.8±13.4 years).

## Methods

DNA was extracted from peripheral venous blood with the iPrep PureLink^®^ gDNA™ Blood Kit (Invitrogen, USA) and iPrep™ purification instrument (Invitrogen, USA).

KIR genotyping was performed by PCR-SSP methods (Olerup SSP™KIR and KIR/HLA Ligand Kit, Sweden) according to the manufacturer’s instructions. In brief, 24 locus-specific primer sets in the KIR genotyping kit allow detection of 16 KIR genes and pseudogenes and discrimination of KIR2DL5A, KIR2DL5B, and KIR3DL1*004 alleles and both groups of KIR2DS4 alleles (KIR2DS4*001 from KIR2DS4*003/004/006/007). The typing was interpreted with the worksheet provided with the kit. KIR/HLA ligands were determined as previously described [9].

### Statistical Analysis

Individual KIR genes, KIR HLA class I ligands, and KIR/HLA class I ligand combination frequencies were determined by direct counting. KIR haplotypes and genotypes were defined in accordance with the Allele Frequency Net Database [20]. Subsequently, individual KIR genotype frequencies were also determined by direct counting. The established frequencies of each of the factors studied were compared between patients and healthy controls using the Pearson chi-square test and Fisher exact test. Odds ratios (ORs) with 95% confidence interval (CIs) were assigned to variables with significant differences determined at a threshold of p<0.05. All statistical analyses were performed with SPSS 16.0 for Windows (SPSS Inc., Chicago, IL, USA).

## Results

### KIR Gene/Pseudogene Frequencies

Overall, no differences in KIR gene/pseudogene frequencies were found between the patients and the healthy individuals, except for a higher incidence of KIR2DS4*001 in the leukemia group (36.6% vs. 20.2%, p=0.017) and in the AML subgroup compared to the control group (52.9% vs. 20.2%, p=0.01) (Table 1). AML patients differed from healthy individuals in the distribution of two other KIR alleles: KIR2DL5A (17.6% vs. 47.7%, p=0.02) and KIR3DS1 (17.6% vs. 47.6%, p=0.02).

The frequency of individual KIR genes and the comparison between the patients and healthy controls are presented in Table 1.

To determine and analyze whether there were findings specific to a particular leukemia type, the KIR frequencies were compared between patients with ALL, AML, and CML. The applied intragroup analysis did not show appreciable differences in the distribution of any KIRs (data not shown).

### KIR Profiles

Thirty-seven different genotypes were determined according to the presence/absence of individual KIRs among the patients and healthy controls, whose characteristics and frequency are presented in Table 2. The comparison between the two groups showed a tendency for a lower frequency of KIR genotype ID2 (6.1% vs. 13.5%, p=0.09; OR 0.4 [95% CI: 0.1-1.3]) in the patient group.

### KIR HLA Class I Ligands

KIR HLA-C ligands were determined in 124 healthy controls: HLA-BBw4 in 113, HLA-ABw4 in 95, and HLA-A3/11 in 83 (Table 3). No significant differences were observed, except for the more frequent presence of homozygous HLA-BBw4 (two HLA-B alleles with Bw4 epitope) in myeloid leukemia patients compared to healthy controls (30.0% vs. 12.5%, p=0.042, OR 3.15 [95% CI: 1.08-9.16]). Considering which amino acid (isoleucine or threonine) was present at position 80 of the HLA-Bw4 molecule, the KIR HLA-B ligand genotype HLA-Bw4Thr80 Thr80was significantly more prevalent in CML and AML compared to the control group (16.7% CML, p=0.04, OR 16.4 [95% CI: 1.0-5.07] and 13.0% AML, p=0.01 OR 12.3 [95% CI: 1.0-3.24] vs. 1.2% in healthy controls). Subgroup analysis based on leukemia type showed no differences (data not shown).

### KIR/HLA Class I Ligand Combinations

The frequencies of individual KIR/HLA class I ligand combinations of inhibitory receptor with the appropriate ligand and the activating counterparts are presented in Table 4. A significantly higher incidence of the KIR3DL2(+)/HLA-A3/11(-) genotype was found in the myeloid leukemia group compared to the healthy control group (AML vs. controls: 88.2% vs. 65.9%, p=0.047, CML vs. controls: 83.3% vs. 65.9%, p=0.047). A lower frequency of KIR3DS1(+)/HLA-ABw4(-) (10.0%, p=0.009) and KIR3DS1(+)/HLA-BBw4(-) (3.3%, p=0.045) combinations among myeloid leukemia cases compared to controls (34.8% and 16.8%, respectively) was observed. In addition, the intragroup analysis between different types of leukemia showed that AML was distinguished from the immunophenotypically opposite group of ALL by the frequency of KIR3DS1(+)/HLA-ABw4(-) (AML versus ALL: 10.0% vs. 29.4%, p=0.007, data not shown).

In the next step, the KIR/HLA class I ligand combinations were investigated taking into account the ligand and the combination of its appropriate inhibitory KIR and activating KIR counterpart (inhibitory KIR/activating KIR/HLA class I ligand). Significant differences were not found between patients and healthy controls in this assay (data not shown). The subgroup analysis, depending on the cytological variant of leukemia, also showed no differences (data not shown).

## Discussion

Particular KIRs and KIR HLA class I ligand polymorphisms associated with a variety of tumors have been reported but the precise disease-predisposing mechanisms have not been elucidated [20].

The KIR2DS4*001 allele was found to be significantly more frequent in the leukemia group compared to healthy controls with the difference being more prominent for AML. Two additional KIRs were identified as protective for AML: KIR2DL5A and KIR3DS1. The protective effect of KIR3DS1 that we established supports the hypothesis that genetic imbalance between activating and inhibitory KIRs in the direction of decreased activation/increased inhibition may contribute to tumorigenesis. These results are in line with the data from a similar disease-associated study in AML patients from Iran [23], as well as its reported protective effect associated with solid tumors [24] and Hodgkin’s disease [25]. However, this hypothesis cannot explain the lower incidence of inhibitory KR2DL5A and the higher incidence of KIR2DS4*001 in the AML group, logically associated with decreased inhibitory and increased NK cell-activating function. KIR2DL5 has been found less frequently in patients with oncohematological diseases such as B-cell chronic lymphocyte leukemia [26] and Hodgkin’s lymphoma patients [24]. Similarly, higher incidence of KIR2DS4 associated with leukemia was reported independently by Giebel et al. [27] and Zhang et al. [28] for CML and Misra et al. [29] for childhood ALL. A similar inconsistency is known for a number of other activating KIRs, which are associated with a higher risk of oncohematological diseases, such as KIR2DS1 [29,30,31], KIR2DS3 [29,31], and KIR2DS2 and KIR2DS5 [29]. On the other hand, the lack of accurate information on the ligand specificity of most KIRs, such as KIR2DL5, and their importance in the regulation of NK cell function significantly impedes the interpretation of the current results. Furthermore, NK cell activity is regulated not only by the individual KIR genes but also by their individual KIR and KIR/HLA class I ligand genotype combinations.

The complex influence of the inherited KIR genes in individual KIR genotypes for development of hematological malignancies was demonstrated first by Verheyden et al. [32]. Their group showed an increased risk of leukemia associated with KIR genotypes, associated with a higher number of inhibitory KIRs. Data from more recent studies that reported predisposition to leukemia associated with KIR genotypes containing a higher number of inhibitory than activating KIRs support this hypothesis [26,33]. A tendency for higher incidence of KIR profile ID2 was found in a study comparing healthy individuals with leukemia patients. The KIR genotype ID2 is characterized by the absence of the inhibitory KIR2DL2 and its activating counterpart, KIR2DS2. The same KIRs, KIR2DL2 and KIR2DS2, are reported as risk factors for acute leukemia [29,33]; in other words, their absence in the KIR genotype ID2 can be interpreted as absence of a genetically predetermined disease susceptibility factor and higher tumor resistance.

Analysis of disease susceptibility by testing the inherited KIRs ligands showed a higher incidence of the HLA-BBw4 (HLA-BBw4/Bw4) homozygous genotype in patients compared to healthy controls. Particularly at risk were patients in the myeloid leukemia group. HLA-BBw4 isa ligand for both KIR3DL1 and KIR3DS1, the former binding it with greater affinity than its activating counterpart. Moreover, KIR3DL1 is significantly more frequent than KIR3DS1. It can be assumed that the expression of HLA-Bw4 ligands maintains NK cells in a state of hyporesponse rather than contributing to NK activation by KIR3DS1/HLA-BBw4. Thus, HLA-BBw4/Bw4 appears to be a risk factor for leukemia development. Two independent studies by Middleton et al. [34] and de Smith et al. [35] also reported homozygous HLA-BBw4 as a risk factor for AML and CML development. de Smith et al. [35] also showed that the KIR3DL1+/HLA-BBw4/Bw4 combination was associated with ALL. Additionally, carriers of two HLA-Bw4 alleles with threonine at the 80 position (HLA- Bw4Thr80 Thr80) were found with a higher frequency in myeloid leukemia cases compared to healthy controls. Bw4Thr80 binds KIR3DL1 with a lower affinity than Bw4Iso80, which results in a lower inhibitory signal to the NK cells by HLA-Bw4Thr80 homozygous individuals. Association of the KIR HLA-Bw4 ligand according to amino acid at position 80 was also reported by Shen et al. [36], who demonstrated significantly higher frequencies of HLA-Bw4Iso80 in the prognostically “poor” AML risk group compared to those with “favorable” risk. In contrast to other studies of KIR HLA class I ligands in leukemia, no other differences were observed [36,37].

Analysis of KIR/KIR HLA class I ligand combinations first confirmed that the investigated polymorphic gene systems have the highest importance for myeloid leukemia susceptibility, whereas their role in disease predisposition to ALL could not be confirmed. The KIR3DL2(+)/HLA-A3/11(-) combination was found significantly more frequently in myeloid leukemia patients than in the healthy population. KIR3DL2 belongs to the framework of KIR genes and is present almost ubiquitously [20]. Thus, the myeloid leukemia-associated genotype KIR3DL2(+)/HLA-A3/11(-) very likely indicates lack of NK cell activity mediated by the inhibitory receptor. Another KIR/HLA class I ligand combination where the activating KIR3DS1 is expressed, but not its putative HLA-A/BBw4 ligand, KIR3DS1(+)/L(-), was found at a lower frequency in the myeloid leukemia group compared to the healthy individuals. On the contrary, La Nasa et al. reported an increased risk of Hodgkin’s disease associated with genotype KIR3DS1(+)/HLA-Bw4(-) [38]. It should be noted, however, that in our study, KIR3DS1 was found to be significantly less common among patients with AML, which may be an explanation for the observed differences and logically raises the question of whether KIR3DS1 is an independent protective factor for leukemia development or the receptor-ligand combinations in which it participates are also important. There is support for both hypotheses in the available literature [23,25]. For the second possible mechanism [26,30], the discussions are only addressing the presence of the binding ligand.

In summary, it is obvious that the patient group is distinguished from healthy controls by the presence of both individual KIR genes and some KIR HLA class I ligands and KIR/HLA class I ligand combinations. These data support the hypothesis of the complex influence of various polymorphic gene systems, in particular KIRs and KIR HLA class I ligands, in the genetically regulated NK immune response. On the other hand, the differences found are valid for patients with myeloid leukemia, but not for the ALL group. These results are not unusual considering the higher susceptibility of myeloblastic cells compared to lymphoblastic cells to NK-mediated cytolysis [39,40,41] and the presumed direct involvement of NK cells in the antitumor response in hemoblastosis of myeloid origin.

### Study Limitations

As a limitation of this study, most importantly, the group of patients analyzed was heterogeneous and included three different types of leukemia characterized by different clinical evolution and prognosis. When dividing patients into separate groups, depending on the type of leukemia (ALL, AML, and CML), the number of subjects analyzed in each group was limited, and highly variable factors such as KIR genotypes did not allow the comparison of each subgroup of leukemia with healthy controls.

## Conclusion

The leukemia susceptibility factors we have found confirm the importance of KIR/HLA class I ligand gene systems in NK-mediated antitumor response in patients with myeloid leukemia. The understanding of the mechanisms of their influence on NK cell function remains limited. Interpretation of the results obtained in the context of the hypothesis of different NK cell activity predetermined at the genetic level depending on the inherited inhibitory/activating potential is difficult due to the poorly studied ligand specificity of the KIR genes as well as their functional activity.

## Figures and Tables

**Table 1:**
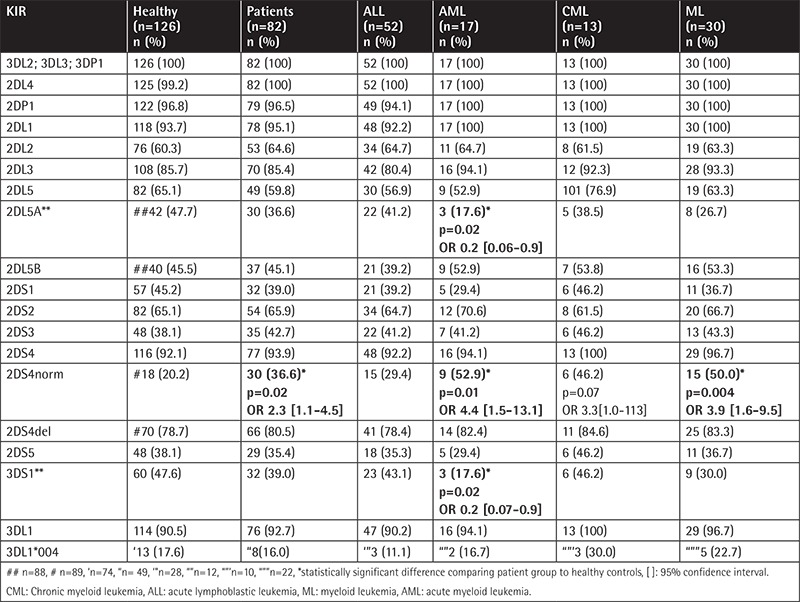
KIR gene frequencies.

**Table 2:**
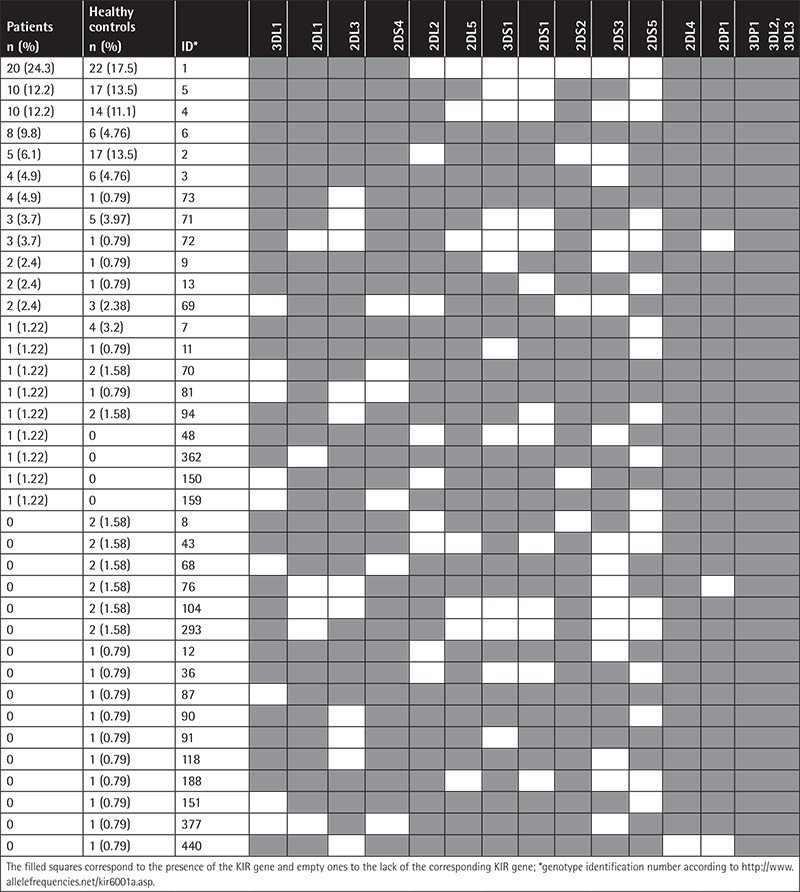
KIR genotype frequencies in patients (n=82) and healthy controls (n=126).

**Table 3:**
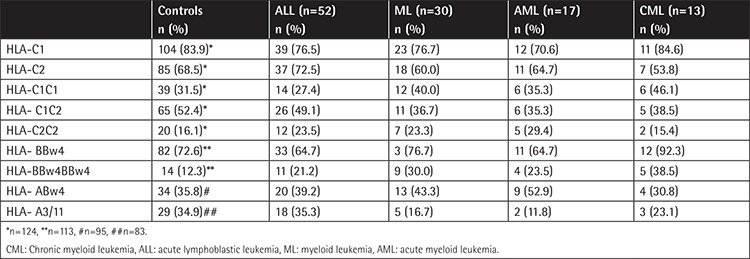
Frequencies of KIR HLA class I ligands and KIR HLA class I ligands combinations.

**Table 4:**
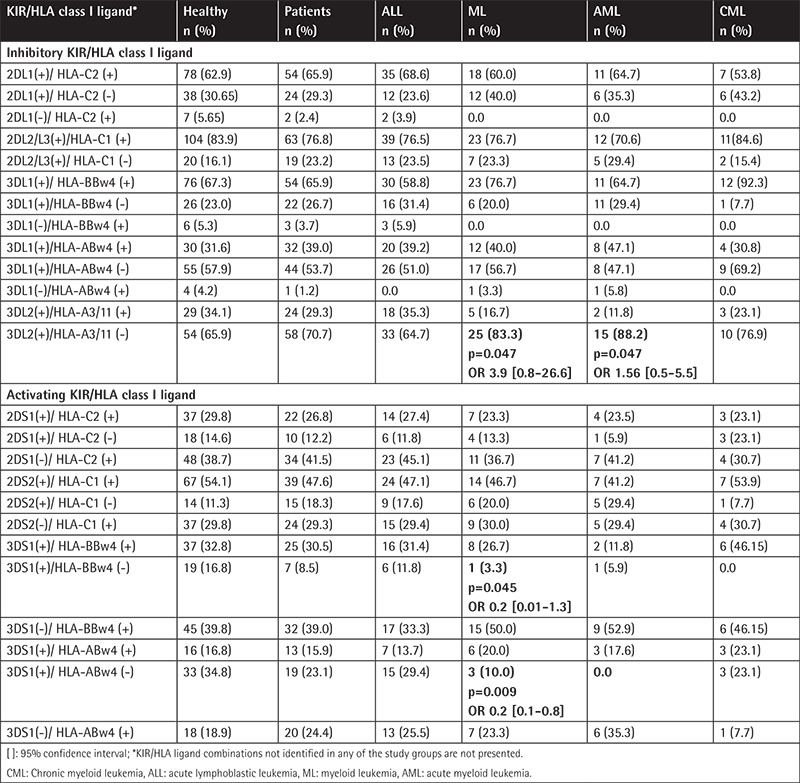
Frequencies of KIR/HLA ligand combinations.

## References

[1] Valerianova Z, Atanasov T, Vukov M. Bulgarian National Cancer Registry. Cancer Incidence in Bulgaria, 2014 & 2015. Available at:.

[2] Trinchieri G (1989). Biology of natural killer cells. Adv Immunol.

[3] Augusto DG (2016). The impact of KIR polymorphism on the risk of developing cancer: not as strong as imagined?. Front Genet.

[4] Parham P (2005). MHC class I molecules and KIRs in human history, health and survival. Nat Rev Immunol.

[5] Collona M, Samaridis S (1995). Cloning of immunoglobulin-superfamily members associated with HLA-C and HLA-B recognition by human natural killer cells. Science.

[6] Winter CC, Long EO (1997). A single amino acid in the p58 killer cell inhibitory receptor controls the ability of natural killer cells to discriminate between the two groups of HLA-C allotypes. J Immunol.

[7] Cella M, Longo A, Ferrara GB, Strominger JL, Colonna M (1994). NK3-specific natural killer cells are selectively inhibited by Bw4-positive HLA alleles with isoleucine 80. J Exp Med.

[8] Gumperz J, Litwin V, Philips JH, Lanier LL, Parham P (1995). The Bw4 public epitope of HLA-B molecules confers reactivity with natural killer cell clones that express NKB1, a putative HLA receptor. J Exp Med.

[9] https://www.ebi.ac.uk/ipd/kir.

[10] Stewart CA, Laugier-Anfossi F, Vély F, Saulquin X, Riedmuller J, Tisserant A, Gauthier L, Romagné F, Ferracci G, Arosa FA, Moretta A, Sun PD, Ugolini S, Vivier E (2005). Recognition of peptide-MHC class I complexes by activating killer immunoglobulin-like receptors. Proc Natl Acad Sci USA.

[11] Foley B, De Santis D, Lathbury L, Christiansen F, Witt C (2008). KIR2DS1-mediated activation overrides NKG2A-mediated inhibition in HLA-C C2-negative individuals. Int Immunol.

[12] Chewning JH, Gudme CN, Hsu KC, Selvakumar A, Dupont B (2007). KIR2DS1-positive NK cells mediate alloresponse against the C2 HLA-KIR ligand group in vitro. J Immunol.

[13] David G, Djaoud Z, Willem C, Legrand N, Rettman P, Gagne K, Cesbron A, Retière C (2013). Large spectrum of HLA-C recognition by killer Ig-like receptor (KIR)2DL2 and KIR2DL3 and restricted C1 specificity of KIR2DS2: dominant impact of KIR2DL2/KIR2DS2 and KIR2D NK cell repertoire formation. J Immunol.

[14] Normam PJ, Abi-Rached L, Gendzekhadze K, Korbel D, Gleimer M, Rowley D, Bruno D, Carrington CV, Chandanayingyong D, Chang YH, Crespí C, Saruhan-Direskeneli G, Fraser PA, Hameed K, Kamkamidze G, Koram KA, Layrisse Z, Matamoros N, Milà J, Park MH, Pitchappan RM, Ramdath DD, Shiau MY, Stephens HA, Struik S, Verity DH, Vaughan RW, Tyan D, Davis RW, Riley EM, Ronaghi M, Parham P (2007). Unusual selection on the KIR3DL1/S1 natural killer cell receptor in Africans. Nat Genet.

[15] Vales-Gomez M, Reuburn H, Mandelboim M, Strominger JL (1998). Kinetics of interaction of HLA-ligands with natural killer cell inhibitory receptors. Immunity.

[16] Pende D, Biassoni R, Cantonini C, Verdiani S, Falco M, di Donato C, Accame L, Bottino C, Moretta A, Moretta L (1996). The natural killer cell receptor specific for HLA-A allotypes - A novel member of the p58-p70 family of inhibitory receptors that is characterized by three immunoglobulin-like domains and is expressed as a 140 kD disulfide-linked dimer. J Exp Med.

[17] Döhring C, Scheidegger D, Samaridis J, Cella M, Colonna M (1996). A human killer inhibitory receptor specific for HLA-A1,2. J Immunol.

[18] Demanet C, Mulder A, Deneys V, Worshman MJ, Class FH, Ferrone S (2004). Down-regulation of HLA-A and HLA-Bw6, but not HLA-Bw4, allospecificities in leukemic cells: an escape mechanism from CTL and NK attack?. Blood.

[19] Verheyden S, Bernier M, Damanet C (2004). Identification of natural killer cell receptor phenotypes associated with leukemia. Leukemia.

[20] http://www.allelefrequencies.net.

[21] Ruggeri L, Capanni M, Casucci M, Volpi I, Tosti A, Perruccio K, Urbani E, Negrin RS, Martelli MF, Velardi A (1999). Role of natural killer cell alloreactivity in HLA-mismatched hematopoietic stem cell transplantation. Blood.

[22] Verheyden S, Scots R, Duquet W, Demanet C (2005). A defined donor activating natural killer cell receptor genotype protects leukemia relapse after related HLA-identical hematopoietic stem cell transplantation. Leukemia.

[23] Shahsavar F, Tajik N, Entezami KZ, Fallah Radjabzadeh M, Asadifar B, Alimoghaddam K, Ostadali Dahaghi M, Jalali A, Ghashghaie A, Ghavamzadeh A (2010). KIR2DS3 is associated with protection against acute myeloid leukemia. Iran J Immunol.

[24] Ozturk OG, Gun FD, Polat G (2012). Killer cell immunoglobulin-like receptor genes in patients with breast cancer. Med Oncol.

[25] Besson C, Roetynck S, Williams F, Orsi L, Amiel C, Lependeven C, Antoni G, Hermine O, Brice P, Ferme C, Carde P, Canioni D, Brière J, Raphael M, Nicolas JC, Clavel J, Middleton D, Vivier E, Abel L (2007). Association of killer cell immunoglobulin-like receptor genes with Hodgkin lymphoma in a familial study. PLoS One.

[26] Karabon L, Jedynak A, Giebel S, Wołowiec D, Kielbinski M, Woszczyk D, Kapelko-Slowik K, Kuliczkowski K, Frydecka I (2011). KIR/HLA gene combinations influence susceptibility to B-cell chronic lymphocytic leukemia and the clinical course of disease. Tissue Antigens.

[27] Giebel S, Nowak I, Wojnar J, Krawczyk-Kulis M, Holowiecki J, Kyrcz-Krzemien S, Kusnierczyk P (2008). Association of KIR2DS4 and its variant KIR1D with leukemia. Leukemia.

[28] Zhang Y, Wang B, Ye S, Liu S, Shen T, Teng Y, Qi J (2010). Killer cell immunoglobulin-like receptor gene polymorphisms in patients with leukemia: possible association with susceptibility to the disease. Leuk Res.

[29] Misra MK, Prakash S, Moulik NR, Kumar A, Agrawal S (2016). Genetic associations of killer immunoglobulin like receptors and class I human leukocyte antigens on childhood acute lymphoblastic leukemia among north Indians. Hum Immunol.

[30] Pamuk GE, Tozkir H, Uyanik MS, Gurkan H, Duymaz J, Pamuk ON (2015). Natural killer cell killer immunoglobulin-like gene receptor polymorphisms in non-Hodgkin lymphoma: possible association with clinical course. Leuk Lymphoma.

[31] Sullivan EM, Jeha S, Kang G, Cheng C, Rooney B, Holladay M, Bari R, Schell S, Tuggle M, Pui CH, Leung W (2014). NK cell genotype and phenotype at diagnosis of acute lymphoblastic leukemia correlate with postinduction residual disease. Clin Cancer Res.

[32] Verheyden S, Bernier M, Damanet C (2004). Identification of natural killer cell receptor phenotypes associated with leukemia. Leukemia.

[33] Varbanova V, Naumova E, Mihaylova A (2016). Killer-cell immunoglobulin-like receptor genes and ligands and their role in hematologic malignancies. Cancer Immunol Immunother.

[34] Middleton D, Diler A, Meenagh A, Sleator C, Gourraud PA (2009). Killer immunoglobulin-like receptors (KIR2DL2 and/or KIR2DS2) in presence of their ligand (HLA-C1 group) protect against chronic myeloid leukaemia. Tissue Antigens.

[35] de Smith AJ, Walsh KM, Ladner MB, Zhang S, Xiao C, Cohen F, Moore TB, Chokkalingam AP, Metayer C, Buffler PA, Trachtenberg EA, Wiemels JL (2014). The role of KIR genes and their cognate HLA class I ligands in childhood acute lymphoblastic leukemia. Blood.

[36] Shen M, Linn YC, Ren EC (2016). KIR-HLA profiling shows presence of higher frequencies of strong inhibitory KIR-ligands among prognostically poor risk AML patients. Immunogenetics.

[37] Babor F, Manser AR, Fischer JC, Scherenschlich N, Enczmann J, Chazara O, Moffett A, Borkhardt A, Meisel R, Uhrberg M (2014). KIR ligand C2 is associated with increased susceptibility to childhood ALL and confers an elevated risk for late relapse. Blood.

[38] La Nasa G, Greco M, Littera R, Oppi S, Celeghini I, Caria R, Lai S, Porcella R, Martino M, Romano A, Di Raimondo F, Gallamini A, Carcassi C, Caocci G (2016). The favorable role of homozygosity for killer immunoglobulin-like receptor (KIR) A haplotype in patients with advanced-stage classic Hodgkin lymphoma. J Hematol Oncol.

[39] Giebel S, Locatelli F, Lamparelli T, Velardi A, Davies S, Frumento G, Maccario R, Bonetti F, Wojnar J, Martinetti M, Frassoni F, Giorgiani G, Bacigalupo A, Holowiecki J (2003). Survival advantage with KIR ligand incompatibility in hematopoetic stem cell transplantation from unrelated donors. Blood.

[40] Venstrom JM, Goolez TA, Spellman S, Pring J, Malkki M, Dupont B, Petersdorf E, Hsu KC (2010). Donor activating KIR3DS1 is associated with decreased acute GvHD in unrelated allogeneic hematopoietic stem cell transplantation. Blood.

[41] Miller JS, Cooley S, Parham P, Farag SS, Verneris MR, McQueen KL, Guethlein LA, Trachtenberg EA, Haagenson M, Horowitz MM, Klein JP, Weisdorf DJ (2007). Missing KIR ligands are associated with less relapse and increased graft-versus-host disease (GVHD) following unrelated dodnor allogeneic HCT. Blood.

